# Nonlinear inversion of electrical resistivity sounding data for multi-layered 1-D earth model using global particle swarm optimization (GPSO)

**DOI:** 10.1016/j.heliyon.2023.e16528

**Published:** 2023-05-23

**Authors:** Kehinde D. Oyeyemi, Ahzegbobor P. Aizebeokhai, Chukwuemeka S. Ukabam, Olusola T. Kayode, Abayomi A. Olaojo, Mohamed Metwaly

**Affiliations:** a Applied Geophysics Programme, Department of Physics, Covenant University, Nigeria; b Canadian Center for Raw Material Display Inc., Saskatchewan, Canada; c Department of Earth Sciences, Ajayi Crowther University, Oyo, Nigeria; d Department of Archaeology, College of Tourism and Archaeology, King Saud University, Riyadh, Saudi Arabia

**Keywords:** Electrical resistivity, PSO, Inversion, Vertical electrical sounding, Geophysical modelling

## Abstract

Interpreting geophysical data requires solving nonlinear optimization problem(s) in inversion. Analytical methods such as least-square have some intrinsic limitations, which include slow convergence and dimensionality, making heuristic-based swarm intelligence a better alternative. Large-scale nonlinear optimization problems in inversion can be solved effectively using a technique within the swarm intelligence family called Particle Swarm Optimization (PSO). This study evaluates the inversion of geoelectrical resistivity data with global particle swarm optimization (GPSO). We attempted to invert field vertical electrical sounding data for a multi-layered 1-D earth model using the developed particle swarm optimization algorithm. The result of the PSO-interpreted VES data was compared with that of the least square inversion result from Winresist 1.0. According to the PSO-interpreted VES results, satisfactory solutions may be attained with a swarm of 200 or fewer particles, and convergence can be reached in fewer than 100 iterations. The GPSO inversion approach has a maximum capacity of 100 iterations, more than the least square inversion algorithm of the Winresist, which has a maximum capacity of 30 iterations. The misfit error of GPSO inversion is $6.14\times 10^{-7}$, much lower than that of the least square inversion of 4.0. The GPSO inversion model has lower and upper limit values of the geoelectric layer parameters model to fit the true model better. The limitations of the developed PSO inversion scheme include a slower execution time of the inversion procedures than the least-square inversion. There is a need for a priori knowledge of the number of layers from borehole reports in the study area. The PSO inversion scheme, however, estimates inverted models closer to the true solutions with greater accuracy than the least-square inversion scheme.

## 1 Introduction

Electrical resistivity sounding or vertical electrical sounding (VES) is a geophysical method used to investigate the subsurface electrical resistivity distribution of the earth. It is based on measuring electrical potential differences between electrodes inserted into the ground. The technique has several areas of applications ranging from subsurface geological evaluation, hydrological evaluation, engineering, and environmental studies [1,2,23,[28], [29], [30],^33^,^46^]. In mineral exploration, the VES technique is used to evaluate the subsurface distribution of metallic and non-metallic deposits [23,46]. This technique is applicable for locating and mapping groundwater resources and assessing the water quality within the delineated aquifers. This information is crucial to exploring, developing and managing water resources [1,2,23,[28], [29], [30]]. The method can also investigate the presence and movement of contaminant plumes within the polluted soil and groundwater [23,33,46]. It can provide information about the depth and extent of contamination and help plan remediation strategies. VES is used in geotechnical engineering to evaluate subsurface soil and rock conditions regarding their strengths and stability. It provides essential information for designing and constructing dams, bridges and buildings [23,33,46]. Generally, vertical electrical sounding is a versatile geophysical technique that provides valuable information about subsurface geology, which can be used in the planning and managing of natural sources, infrastructural development, and environmental protection.

Inversion of the vertical electrical sounding data using a least square inversion scheme has revealed an intrinsic link between apparent resistivity data and the parameters of a multi-layered earth model [45]. The probabilistic nature of the global optimization techniques has made them a better alternative for an efficient sample of model space. The global optimization techniques are very robust as they can resolve inverse problems as though they were simple problems. Their inherent limitation is related to their dimension issue and the need for high computational costs towards solving forward problems (predictions) (Mohammadzadeh and Gharehchopogh, 2020). The inverse problem aims to search for the best parameter estimates to minimise the numerical disparity between the expected outputs and measurements with all known constants being satisfied. According to Ref. [37]; this is an optimization problem. To improve the quality of the estimated parameters, a wide range of credible prior information on the structures, subsurface geology, and geophysical data should be combined. Many available methods have been developed over the years for model parameter estimation. They include conjugate gradient, ridge regression, and steep descent techniques, generally termed the local search method. Also, the genetic algorithm and simulated annealing techniques are termed the global search methods. If any apriori information is lacking, local optimization techniques can generate unpredictable outcomes [16]. investigated the saltwater intrusion problem with global optimization algorithms. Traditionally, linearised or least-squares inversions focus on linearising the model response mapping. This technique has been reported for several applications in the inversions of electrical resistivity and induced polarization data [1,2,13,24,27,30,36].

Metaheuristic algorithms have been modified to solve many optimization problems of industrial, scientific, and engineering applications. A genetic algorithm was used to improve the performance of the cuckoo search optimization technique [43]. [19] proposed improved methods on the Harris Hawks Optimization technique for social network evaluation. Several techniques involving integrating quantum computing concepts into metaheuristic algorithms to obtain quantum computing-inspired metaheuristic algorithms with higher speed, accuracy, and performance have been reviewed by Ref. [20] based on their applications in science and engineering. A comprehensive review of applications and recent advances in the sparrow search algorithm was reported by Ref. [21]. Shear-wave velocity log data were predicted from conventional wireline logs data from an offshore oil field in Western Australia using three different developed metaheuristic algorithms [26].

Moreover, theoretical frameworks of several swarm-based metaheuristic optimization techniques were reviewed by Ref. [35]. Metaheuristic algorithms including Generic and Simulated annealing algorithms are more suitable global optimization methods when considering the large degree of freedom in the inversion of geophysical data, incomplete data, and when the existing solutions are either non-unique or multiple. There are numerous examples of the applications of these algorithms in inversions of geophysical data [[5], [14], [15], [16], [17], [25], [34], [38], [39], [40]]. Simulated Annealing was used to invert 2D electrical resistivity tomography by Ref. [7]; and [5] applied Simulated Annealing to tackle the issue of uncertainty, resolution, and sensitivity in the inversion of induced polarization data and vertical electrical sounding (VES) data over a multi-layered 1D layer model [14]. used a binary genetic technique for the inversion of geoelectrical sounding data, and [8] inverted self-potential data using a hybrid genetic price algorithm. Model parameters estimations from the residual gravity anomaly of idealised geometrical bodies have been evaluated with the use of a simulated annealing global optimization algorithm [4], differential evolution algorithm [9], and global particle swamp optimization algorithm [42]. Magnetic anomalies source parameters have also been estimated using the optimization of the ant colony algorithms [6,44] and particle swamp optimization [[10], [11], [12]]. [47] improved the convergence rate and the performance PSO algorithm using the backtracking search algorithm (BSA).

This study uses a particle swamp optimization algorithm to interpret electrical resistivity sounding data. We chose the VES geophysical data sets to investigate the distribution of subsurface resistivity within a multilayered 1D earth model for us to tune the parameters of the global particle swarm optimization such that a convergence of several possible solutions within a search space can be achieved. The objective is to compute the best parameter to minimise the differences between the expected outputs and actual measurements. The findings show that PSO-interpreted VES fits the true model more accurately because it gets up to about 100 iterations better than the conventional least-square inversion scheme of the WinResist computer program with a maximum of 30 iterations. The misfit error of the GPSO inversion scheme is much lower than the traditional least-square inversion scheme.

## 2 Method

### 2.1 Inversion of geoelectrical sounding data

The inversion of the Vertical Electrical Sounding (VES) model assumes that the terrain is stratified horizontally with resistivity and thickness values $\rho_{k}$ and $t_{k}$ of the kth electrical layers. The subsurface parameter can be represented by a vector given as $m=\left(\rho_{1},t_{1},\rho_{2},t_{2},\rho_{3},t_{3},...,\rho_{n-1},t_{n-1},\rho_{n}\right)$ belonging to a 2n−1 dimensional vector space m, where n is the number of layers. The assumption of the forward model problem is that m is known while corresponding apparent resistivity ($\rho_{a}^{*}\left(s,m\right)$) at any desired surface locations from the measured data is predicted. The forward problem can be mathematically expressed as:

(1) $$\rho_{a}^{*}\left(s.m\right)=\rho_{1}+s^{2}\int_{0}^{\infty }\left(T_{1}\left(\lambda ,m\right)-\rho_{1}\right)\cdot J_{1}\left(\lambda s\right)\lambda d\lambda$$

Bessel function, resistivity transform kernel for layer 1, and the integration variables are represented as $J_{1}$, $T_{1}$, and λ. The computation of the resistivity transform kernel $T_{i}\left(\lambda \right)$ for each ith layer in a recursive manner from the last to the first layer is given by Pekeris relations [31] as:

(2) $$\begin{array}{l} T_{n}\left(\lambda \right)=\rho_{n}\\ T_{i}\left(\lambda \right)=\frac{\left[T_{i+1}+\rho_{i}\cdot th\;\left(\lambda t_{i}\right)\right]}{\left[1+T_{i+1}\;\tanh\left(\lambda t\right)/\rho_{i}\right]} \end{array}$$

Equation (1) is computed using the convolution theorem, while the inverse problem involves the computation of model parameters m with the available observed data given as:

$\rho_{a}^{0}\left(s\right)=\left(\rho_{a}^{0}\left(s_{1}\right),\rho_{a}^{0}\left(s_{2}\right),...\rho_{a}^{o}\left(s_{nd}\right)\right)\text{,}$ for an array $s=\left(s_{1},s_{2},...,s_{nd}\right)$

Where $\rho_{a}^{0}$ and s are the measured apparent resistivity and distances half of the current electrode spacing between $\frac{AB}{2}$, respectively. This represents the apparent resistivity curve with due consideration of the physics of the forward problem in equation (1). To measure the difference between observations and predictions, a relative misfit is used:

(3) $$RE\_RMS\left(m\right)=\frac{{\left\|\rho_{a}^{0}\left(s\right)-\rho_{a}^{*}\left(s,m\right)\right\|}_{2}}{{\left\|\rho_{a}^{0}\left(s\right)\right\|}_{2}}$$

### 2.2 The particle swarm optimization (PSO)

The particle swarm optimization is a stochastic process computation technique used for optimization inspired by individuals' social behaviour (called particles) first introduced by Ref. [22]. It primarily stimulates the conduct of insects, birds and fishes in their quest for food within their natural habitats. These animals represent the particles or models in the PSO algorithm, and each model is described by its position vector that represents the parameters model and a velocity vector. Misfit-function values and the history of the particle's model parameters are used to update the velocity vector of each particle's model [22]. described history as the “knowledge” acquired by each particle, representing a conceptual autobiographical memory of the entire swarm of particles. By returning to the solution space's promising areas that were identified from the iteration history and continuously searching for the best misfit-function values over time, the social behaviour of the swarm is used to compute the update of the misfit-function value for each particle in the swarm that has adapted to its environment.

Mathematically, the value of the ith model parameter will be updated for the jth particle at the iteration k+1 in the swarm as:

(4) $$m_{i,j}^{k+1}=m_{i,j}^{k}+u_{i,j}^{k+1}\Delta t\text{,}$$

The corresponding velocity is represented as $u_{i,j}^{k+1}$ and Δt is the time step value (≈1). Each model parameter has the velocity vector computed as:

(5) $$u_{i,j}^{k+1}=wu_{i,j}^{k}+c_{1}r_{1j}^{k}\frac{\left(P_{i,best}^{k}-m_{i,j}^{k}\right)}{\Delta t}+c_{2}r_{2,j}^{k}\frac{\left(P_{i,best}^{k}-m_{i,j}^{k}\right)}{\Delta t}$$

Where:

$u_{i,j}^{k+1}$: this represents the velocity vector of ith the model parameter for particles in the swarm at iteration k. It denotes the momentum that prevents the particles from abruptly changing their orientation as well as the bias in that direction.

$m_{i,j}^{k}$: this represents the value of the ith model for the jth particle in the swarm at iteration k.

$P_{i,best}^{k}$: this represents the ith model parameter value for the personal best misfit function gained by the jth in the swarm, from the initialisation through to the iteration k.

$P_{i,gbest}^{k}$: this represents ith model parameter value for the global best misfit function achieved by the jth in the swarm, from initialisation to iteration k.

w is the inertia weight, which controls the current velocity vector. It scales the effect of the current velocity vector on the updated velocity. When w is large, It will enlarge the updated velocity vector, forcing the algorithm to broaden its exploration of the solution model space [41]. expressed the variation of w to linearly decrease as:

(6) $$w_{k+1}=w_{\max}-\left(\frac{w_{\max}-w_{\min}}{k_{\max}}\right)k$$

Where $w_{\min}$ and $w_{\max}$ are the maximum and minimum values of inertia weight and $k_{\max}$ is

The maximum iteration numbers, and k is the current iteration number.

$r_{1,j}^{k}$ and $r_{2,j}^{k}$: each of these connotes a random number within the interval [0,1] at iteration k.

$c_{1}$ and $c_{2}$: They stand for the positive acceleration constants that are used to evaluate the relative importance of the social and cognitive characteristics. The stability of the particle swarm optimization that could make the system converge to a local optimum value is dependent on the satisfaction of the following conditions [32]:

(7) $$0<c_{1}+c_{2}<4$$

(8) $$\left(\frac{c_{1}+c_{2}}{2}\right)-1<1$$

**Fig. 1:**
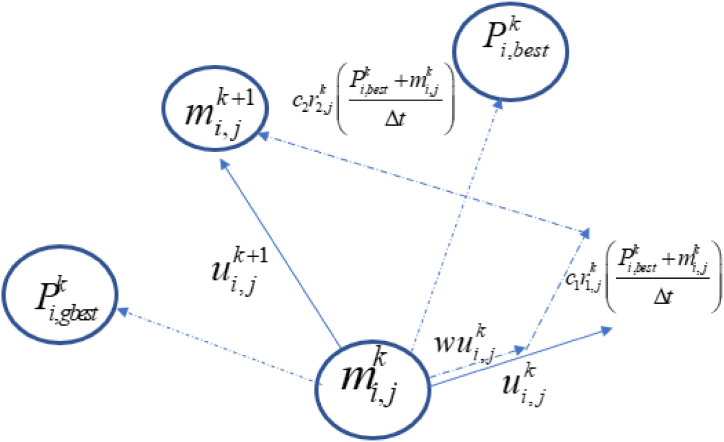
The model parameters for GPSO and velocity updates (Modify after [32]).

**Fig. 2:**
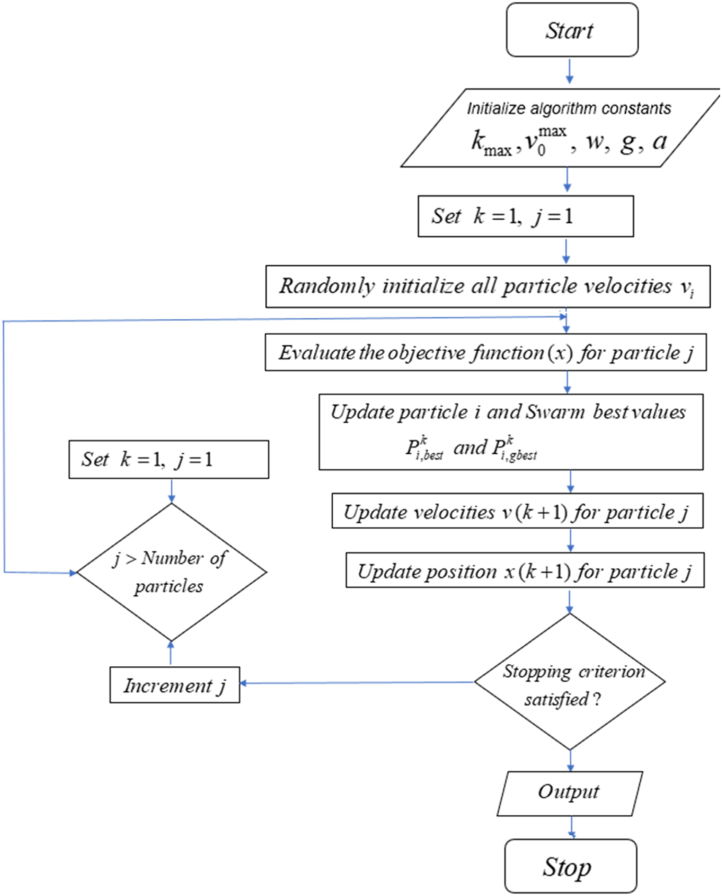
The Asynchronous (nonlinear) Particle Swarm Algorithm flowchart.

In equation (5), the term $c_{1}r_{1,j}^{k}\left(\frac{P_{i,best}^{k}+m_{i,j}^{k}}{\Delta t}\right)$ represents the cognitive component which computes the performance of the particles concerning past performances. The component denotes the individual memory of the position best for the particle. The tendency of particles to revert to places that gave them the most satisfaction in the past is represented by the cognitive component, which is also known as nostalgia of the particle. On the contrary, the term $c_{2}r_{2,j}^{k}\left(\frac{P_{i,gbest}^{k}+m_{i,j}^{k}}{\Delta t}\right)$ represents the social element that calculates how well the particles perform in comparison to a swarm. This element has the effect of making each particle gravitate towards the optimal location within its neighbourhood. Fig. 1 shows the schematic diagram of global particle swarm optimization (GPSO). Equation (4) shows the updating of the model parameter value and the velocity of the swarm of particles. The global and local optimal parameters such as the size of the cognitive parameters $c_{1}$, social parameters $c_{2}$, and inertial weight w influence the updated model parameters [18,39] The GPSO algorithm makes use of the following parameters: swarm magnitude (particles number), acceleration coefficients ($a_{g}$ for global and $a_{l}$ for local), number of iterations (k,k+1,...), initial weights (w), and velocity components ($v_{j}\left(k\right),v_{j}\left(k+1\right)$). The developed global particle swarm optimization (GPSO) algorithm was implemented in MATLAB, and the flowchart for its implementation is shown in Fig. 2. The flowchart for implementing the PSO algorithm to interpret vertical electrical sounding (VES) data includes the following steps:

(i) Input the VES data, including the resistivity values measured at a different point $\frac{AB}{2}$.
(ii) Define the objective function that you want to optimize. This could include a function that calculates the variation between the predicted resistivity values from a given model and the actual measurements.
(iii) Initialize the PSO algorithm by specifying the particles numbers, search space, and initial particles' velocities and positions.
(iv) Compute the objective function for each position of each particle, update the position of the particle, and update the personal best position for each particle and the existing global best position for each particle.
(v) Update the particle's velocities and positions based on the personal and global best positions and some randomness to encourage exploration.
(vi) Repeat steps (iv) and (v) until some stopping criteria such as maximum number of iterations and the convergence of the objective function are satisfied.
(vii) Extract the model parameters that correspond to the global best position discovered by the PSO algorithm. These parameters should provide an excellent fit to the VES data.

The raw vertical electrical sounding (VES) data used to test the efficacy of the GPSO inversion algorithm in Ota, southwestern Nigeria, are presented in Table 1. The datasets were acquired manually using the ABEM Terrameter 4000. The performance of the GPSO inversion scheme was then compared with that of the least square inversion scheme of the Winresist 1.0 computer program [45]. It is worth noting that the quality of the results from implementing the PSO algorithm depends mainly on the chosen objective function and the settings of other parameters. Therefore, careful experimentation and validation are necessary to ensure that the PSO algorithm is used appropriately and effectively for a given task. The availability of prior subsurface geological knowledge from borehole reports is imperative for the use of this technique to interpret vertical electrical sounding data.

**Table 1:** Raw VES data.

AB/2 (m)	1.0	1.3	1.8	2.4	3.2	4.2	4.2	5.5	7.5
$\rho_{app}\left(\Omega m\right)$	81.08	145.2	172.66	205.99	243.85	268.93	269.53	318.04	352.02
AB/2 (m)	10.0	13.0	13.0	18.0	24.0	32.0	42.0	55.0	55.0
$\rho_{app}\left(\Omega m\right)$	393.64	445.42	444.12	527.02	622.83	692.16	690.48	691.85	692.75
AB/2 (m)	75.0	100.0	130.0	130.0	180.0	240.0			
$\rho_{app}\left(\Omega m\right)$	526.68	409.38	383.28	332.78	204.48	125.27			

## 3 Results and discussion

**Fig. 3:**
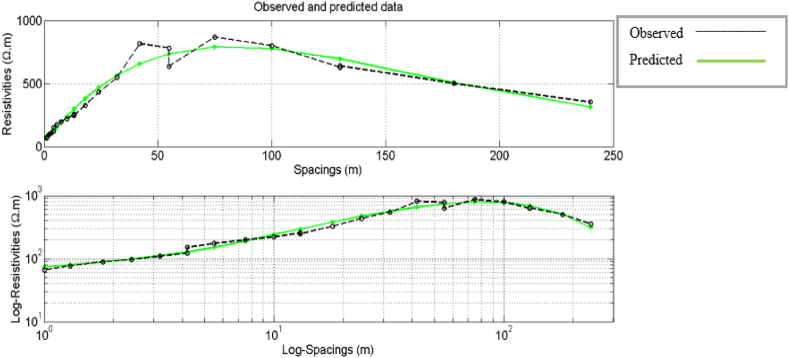
Graph comparing the observed and calculated apparent resistivity values from the best model against half the Current electrode spacing.

**Fig. 4:**
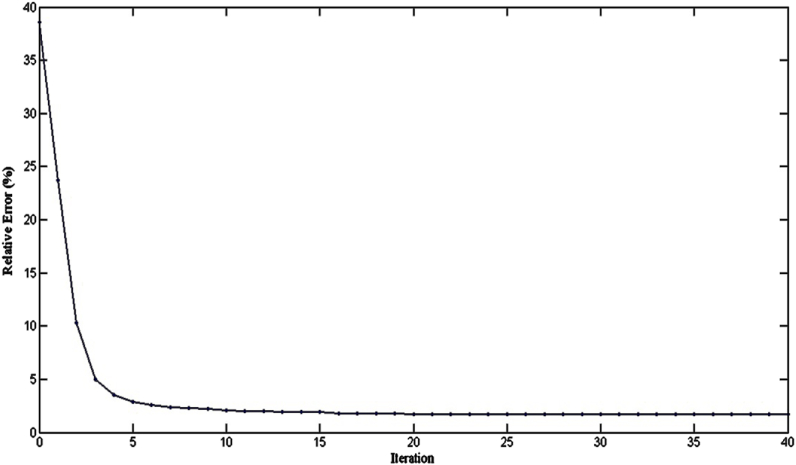
Relative error of PSO inversion scheme in terms of iterations.

**Fig. 5:**
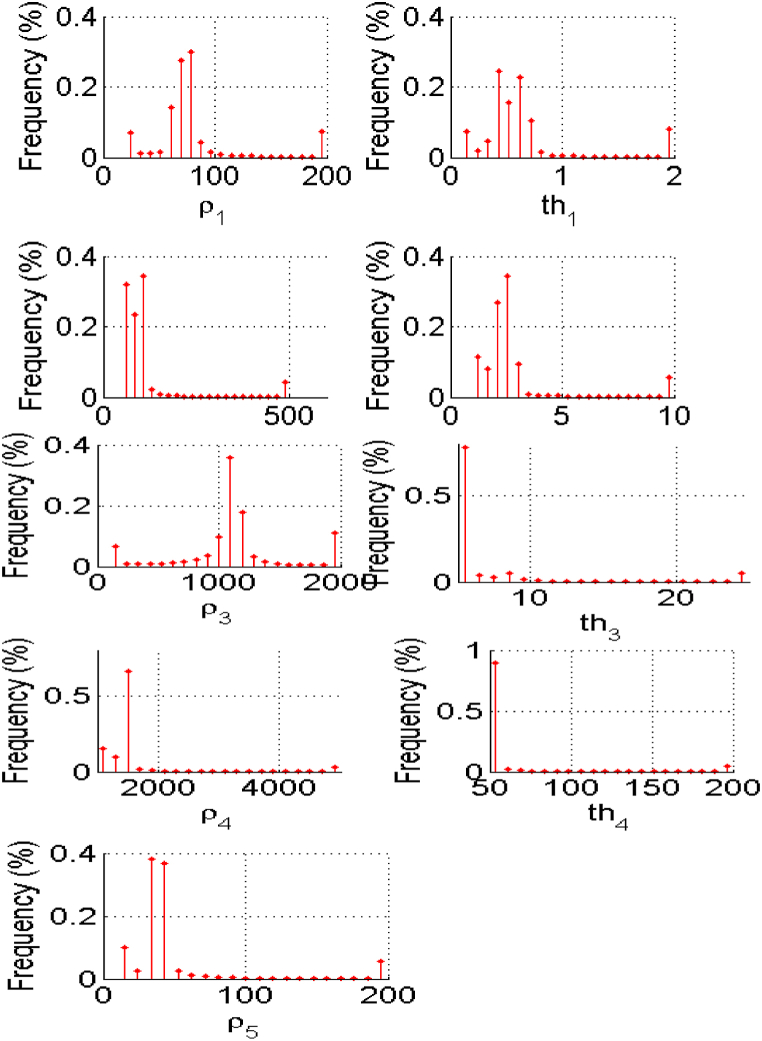
Statistical distributions of resistivities and thicknesses of the five ground layers with an error of less than 1% obtained for a population of 200 particles in 100 iterations.

The PSO-based algorithm was used to invert the VES data in Table 1. Initially, in the algorithm, each particle of a population of the chosen size and the corresponding apriori information is placed (randomly or not) in the search space, a collection of possible solutions. In this case, apriori knowledge of the five subsurface geologic layers (N = 5) from the previous studies and available borehole reports within the area [1,2,29]. The PSO-based inversion algorithm worked with an assumption that the subsurface is horizontally stratified. Also, the lower and upper limits of the values of resistivities and layers thicknesses, as presented in Table 2, were supplied to define the search space by the PSO algorithm. The GPSO inversion scheme of the measured apparent resistivity data (Table 1) employed the lower and upper resistivity and thickness limits (Table 2) to interpret the five delineated geoelectric layers. We chose a swarm of two hundred particles (two hundred models) that evolved, each exploring the search space in 100 iterations. All investigated models can retain only those obtained with a misfit error less than a given value which was chosen to be 1%. There are thus as many times 2N-1 values (here, five resistivity values and four thickness values) that models retained. The results are presented graphically; first is the graph of observed and apparent resistivities from the best model (Fig. 3) according to the half-length between the injection electrodes (Spacing), the graph of the evolution of the relative error as a function of iterations (Fig. 4) and, secondly, the frequency distributions of the thicknesses and resistivities different layers leading to an acceptable model with less than 5% relative error (Fig. 5).

**Table 2:** The upper and lower limits of the resistivity and thickness of the five layers.

	Layer	1	2	3	4	5
Resistivity	Lower limit	20	50	100	1000	10
	Upper limit	200	500	2000	5000	200
Thickness	Lower limit	0.1	1	5	50	–
	Upper limit	2	10	25	200	–

**Fig. 6:**
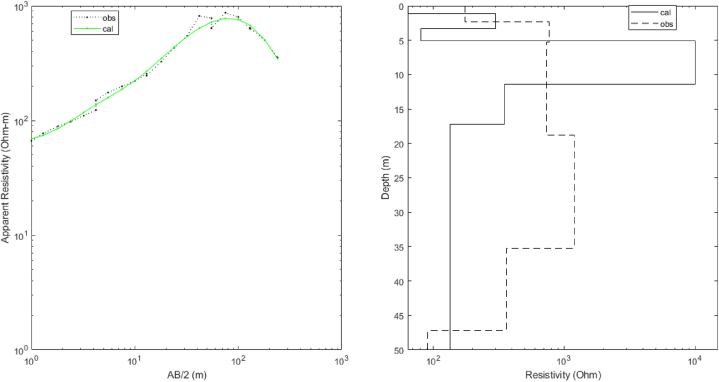
(A) PSO inversion of VES data with the best model at a root mean square error of 6.14.

**Fig. 7:**
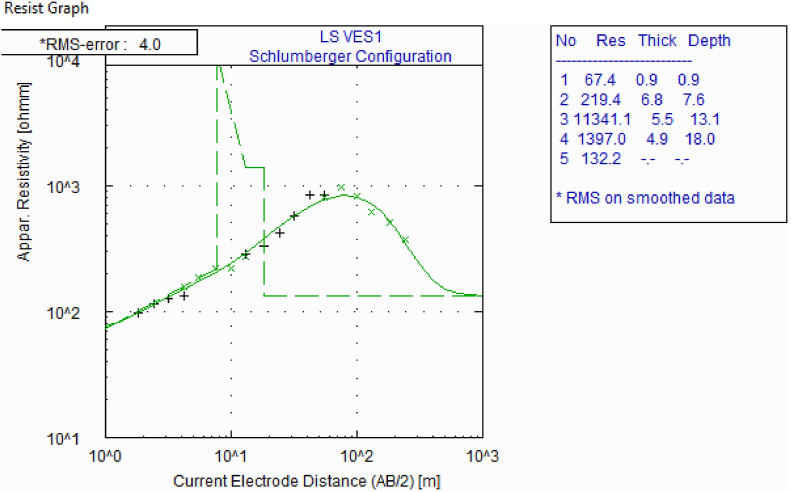
VES interpretation by Winresist 1.0 computer program using the Least-square method with an RMS error of 4.0.

The GPSO inverted VES data result is presented in Fig. 6 with a misfit error $6.14\times 10^{-7}$. To determine the efficacy and accuracy of the PSO algorithm in calculating the resistivities and thicknesses of the subsurface layers (Table 3), we compared the results of the PSO inverted VES with the VES data interpretation using Winresist 1.0 software package. This software package uses a Least-square algorithm for resistivity model inversion of the raw VES data in Table 1 with an RMS error of 4.0% (Fig. 7). Table 4 reveals that thicknesses of subsurface layers from GPSO inversion and Least-square schemes are comparable. Despite the more extended computation and execution time, the misfit error for the GPSO inversion scheme is lower than that of the least-square inversion scheme.

**Table 3:** The subsurface layers' true resistivity, thickness and depth.

Layers	ρ(Ωm)	Thickness (m)	Depth (m)
1	70	1	1
2	166.33	4.2	5.2
3	708.33	18	23.2
4	3060.00	110	133.2
5	42.11	–	–

**Table 4:** Table showing the modelled resistivity, thickness and depth of the ground layers with PSO and Least-Square inversion schemes.

Layers	PSO Inversion			Least Square Inversion		
	ρ(Ωm)	Thickness (m)	Depth (m)	ρ(Ωm)	Thickness (m)	Depth (m)
1	70	1	1	67.44	0.9	0.9
2	166.33	4.2	5.2	219.4	6.8	7.6
3	708.33	18	23.2	11341.1	5.5	13.1
4	3060.0	110	133.2	1391.0	4.9	18.0
5	42.11	–	–	132.2	–	–

Unlike traditional optimization methods, which postulate the existence of a better solution to discover, the global algorithms assume that the model is a designed random vector [3]. So, the solution to an optimization problem by particle swarm will result in model parameters with frequency distributions to the experimental data. Fig. 5 reveals the model parameters of the interpreted VES data in the form of the true resistivities and thicknesses of the subsurface. The PSO algorithm generated a distribution of statistical parameters of the models with respect to the known experimental data f(m|d). Global PSO algorithms, therefore, allow us to have a statistical distribution view of the phenomenon studied, given the discrete number of data, the presence of measurement noise and the limitations of the physical model compared to reality. Applying this method makes it possible to re-analyze archival data with a statistical distribution and less deterministic (Fig. 5), which is preferable in the vertical electrical sounding (VES) technique for which equivalence principles are well known.

The GPSO algorithm inversion scheme for the VES data interpretation applies statistical models with a wide range of choices in form of layers’ resistivities and thicknesses within the search space, which are, in turn, related to the field measured resistivity data. The PSO algorithm scheme has a plug-in for setting the lower and uppermost limits for the resistivity and thickness, with an avenue to iterate the layer models with the field-measured data until the best line of fit is established (Fig. 3). The interpretation of the measured field data in Table 1 with the PSO-based inversion scheme resulted in a misfit error of 6.14 × 10^−7^. In contrast, interpreting the same data with the Least-Square inversion of Winresist 1.0 resulted in RMS error of 4.0. The lower misfit error generated by the PSO-based inversion is a pointer to the fact that there is an agreement between the observed data line and the predicted line. Fig. 4 revealed that the relative error (%) reduces with rise in the number of iterations; the PSO inversion algorithm scheme has a capacity of 100 iterations, whereas the Least-Square procedures of WinResist 1.0 computer program provides for 30 iterations [45]. The more the data are related, the better the line of best fit due to noise removal and signal sustainability. The PSO-based algorithm, having lower and upper limits, cancels out the need for partial curve matching and the generation of an input model for the iteration of true geoelectric layer resistivity and thickness values. This is one of the technical constraints and challenges observed in using Least Square inversion scheme that the PSO inversion algorithm has resolved.

## 4 Conclusion and future works

The current study was done to utilise the GPSO algorithm for the inversion of vertical electrical soundings (VES) data. The primary goal was to implement the global particle swarm optimization algorithm developed in this study to interpret field-acquired VES data. The GPSO algorithm, a random vector model, assumed the subsurface to be horizontally stratified. The equivalence principle in VES was used to adjudicate the resistivities of the geoelectric section with the corresponding thickness. The outputs of the PSO-based inverted VES data were compared with the results of the least-square inversion scheme based on computation time and misfit error. The study findings revealed that the PSO-based VES inversion gives impressive convergence rate curves with low-misfit geophysical models than the least-square inversion scheme utilizing similar initial and search space. GPSO algorithm resolves and bridges the gap between the expected and actual inversion model results better with high resolution. There is a higher convergence rate, fewer misfit errors, and greater iterations using the GPSO inversion scheme compared to the Least-squares inversion model. The comparative assessment of the VES data using GPSO and the Least-square inversion models showed a high degree of similarity in the generated geoelectric layers sequence but a better and higher depth resolution by the GPSO inversion scheme. Despite the slower execution time, the GPSO algorithm inversion scheme can resolve the geoelectrical parameters of the delineated subsurface layers better with fewer misfit errors.

For the inversion of the 2D electrical resistivity imaging (ERI) data, we suggest the modification of the developed GPSO technique in this study. To increase speed, accuracy, and performance, it is advised that the developed GPSO algorithm in this study be combined with the backtrack search algorithm and the quantum computing concept. Additionally, we advise the interpretations of geoelectrical resistivity data utilizing other swarm-based metaheuristic optimization methodologies including artificial bee colony, antlion optimization, shuffled frog leaping optimization, firefly algorithm, cuckoo search algorithm, sailfish optimization, moth flame optimization, squirrel search algorithm, bat algorithm, crow search algorithm, whale optimization algorithm, grey wolf optimization, and bacterial foraging optimization.

## Author contribution statement

Kehinde David Oyeyemi: Mohamed Metwaly: Conceived and designed the experiments; Performed the experiments; Analyzed and interpreted the data; Wrote the paper.

Ahzegbobor P Aizebeokhai: Chukwuemeka S Ukabam: Conceived and designed the experiments; Performed the experiments; Analyzed and interpreted the data.

Olusola T Kayode: Abayomi A. Olaojo: Analyzed and interpreted the data; Wrote the paper.

## Data availability statement

Data included in article/supp. Material/referenced in article.

## Funding statement

This work was funded by the Research Supporting Project Number (RSP2023R89), King Saud University, Riyadh, Saudi Arabia.

## Data available statement

This article presents the data, and the codes are within the supplementary file.

## Additional information

No additional information is available for this paper.

## Declaration of competing interest

The authors declare that they have no known competing financial interests or personal relationships that could have appeared to influence the work reported in this paper. The authors declare the following financial interests/personal relationships which may be considered as potential competing interests.
